# Ecological Effects of the Invasive Giant Madagascar Day Gecko on Endemic Mauritian Geckos: Applications of Binomial-Mixture and Species Distribution Models

**DOI:** 10.1371/journal.pone.0088798

**Published:** 2014-04-30

**Authors:** Steeves Buckland, Nik C. Cole, Jesús Aguirre-Gutiérrez, Laura E. Gallagher, Sion M. Henshaw, Aurélien Besnard, Rachel M. Tucker, Vishnu Bachraz, Kevin Ruhomaun, Stephen Harris

**Affiliations:** 1 School of Biological Sciences, University of Bristol, Bristol, United Kingdom; 2 National Parks & Conservation Service, Reduit, Mauritius; 3 Durrell Wildlife Conservation Trust, Jersey, Channel Islands; 4 Mauritian Wildlife Foundation, Vacoas, Mauritius; 5 Naturalis Biodiversity Center, Leiden, The Netherlands; 6 Institute for Biodiversity and Ecosystems Dynamics (IBED), University of Amsterdam, Amsterdam, The Netherlands; 7 Laboratoire de Biogeographie et d'Ecologie des Vertébrés, Centre d'Ecologie Evolutive et Fonctionnelle, Montpellier, France; 8 Department of Animal and Plant Sciences, University of Sheffield, Sheffield, United Kingdom; Università degli Studi di Napoli Federico II, Italy

## Abstract

The invasion of the giant Madagascar day gecko *Phelsuma grandis* has increased the threats to the four endemic Mauritian day geckos (*Phelsuma* spp.) that have survived on mainland Mauritius. We had two main aims: (i) to predict the spatial distribution and overlap of *P. grandis* and the endemic geckos at a landscape level; and (ii) to investigate the effects of *P. grandis* on the abundance and risks of extinction of the endemic geckos at a local scale. An ensemble forecasting approach was used to predict the spatial distribution and overlap of *P. grandis* and the endemic geckos. We used hierarchical binomial mixture models and repeated visual estimate surveys to calculate the abundance of the endemic geckos in sites with and without *P. grandis*. The predicted range of each species varied from 85 km^2^ to 376 km^2^. Sixty percent of the predicted range of *P. grandis* overlapped with the combined predicted ranges of the four endemic geckos; 15% of the combined predicted ranges of the four endemic geckos overlapped with *P. grandis*. Levin's niche breadth varied from 0.140 to 0.652 between *P. grandis* and the four endemic geckos. The abundance of endemic geckos was 89% lower in sites with *P. grandis* compared to sites without *P. grandis*, and the endemic geckos had been extirpated at four of ten sites we surveyed with *P. grandis*. Species Distribution Modelling, together with the breadth metrics, predicted that *P. grandis* can partly share the equivalent niche with endemic species and survive in a range of environmental conditions. We provide strong evidence that smaller endemic geckos are unlikely to survive in sympatry with *P. grandis*. This is a cause of concern in both Mauritius and other countries with endemic species of *Phelsuma*.

## Introduction

Invasive alien species (IAS) are capable of establishing, dispersing and causing harm to indigenous species [1]. They can cause cascading effects in native ecosystems by disrupting trophic interactions and sharing ecological resources with native species [2]–[4]. Although IAS have led to the extinction of many endemic species [5], they are still being spread around the world. Oceanic islands are at greater risk [6], [7] as island endemics have evolved in the absence of predators and do not have anti-predator defence mechanisms [8]. So successful invaders encounter less competition from island endemics and, being genetically more adaptable, tend to expand their niches [9].

**Figure 1:**
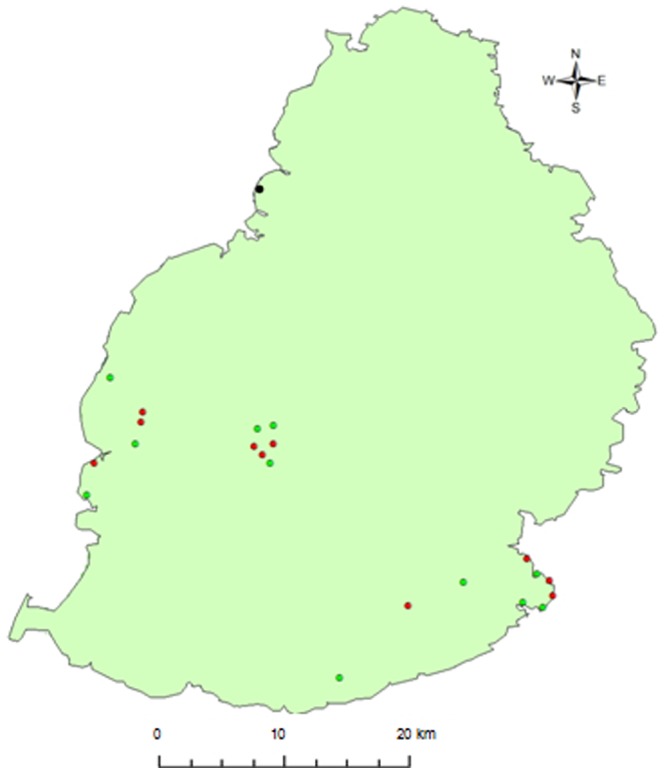
Locations of the ten sites with *Phelsuma grandis* (red) and 11 sites without *Phelsuma grandis* (green) used to estimate the abundance of endemic species of *Phelsuma*. The black dot indicates Baie du Tombeau, the location where *Phelsuma grandis* was first introduced.

Reptile extinctions in Mauritius have mostly been due to the introduction of mammalian invaders, with 69% of its endemic reptile diversity lost since human colonisation at the end of the 16th century [10], [11]. Only one terrestrial skink (*Gongylomorphus bojerii fontenayi*, Macchabé skink) and four arboreal day geckos (*Phelsuma cepediana*, blue-tailed day gecko; *Phelsuma guimbeaui*, lowland forest day gecko; *Phelsuma ornata*, ornate day gecko; *Phelsuma rosagularis*, upland forest day gecko) survive on mainland Mauritius. While *P. cepediana* consists of three un-described species [11], they are treated as a single species for this analysis because they cannot be distinguished phenotypically with confidence. A further seven endemic species of reptile survive on seven offshore islets, one of which is *Phelsuma guentheri* (Günther's gecko), a large species of day gecko that co-existed with most, if not all, of the smaller species of *Phelsuma* on mainland Mauritius before the 1800s [12]. However, the recent introduction of *Phelsuma grandis* (giant Madagascar day gecko) is believed to threaten the four surviving smaller species of *Phelsuma* on mainland Mauritius. *P. grandis* was originally introduced in Baie du Tombeau (Figure 1) in the early 1990s through the pet trade and has since been deliberately moved elsewhere. This species can attain a length of 24–30 cm [13], nearly double the length of the four endemic geckos [14].

Although the export of *P. grandis* is controlled through CITES (Appendix II), it has also been introduced to Afghanistan [15], Florida [16], Hawaii [17] and Réunion [18]. Whether *P. grandis* has significant impacts in these localities is currently unknown, but it has been observed predating the endemic *Phelsuma borbonica* (gecko vert des hauts) and introduced *Gehyra mutilata* (stump-toed gecko) in Réunion [18]. In Mauritius, *P. grandis* has been observed to predate on geckos such as *P. ornata* and the introduced species *G. mutilata*, *Hemidactylus frenatus* (common house gecko) and *Hemidactylus parvimaculatus* (Sri Lankan house gecko).

Apart from the anecdotal sightings of predation and localised presence of *P. grandis* in Mauritius, our current knowledge of its ecological impact is limited. Therefore, we first investigated the potential distribution of the endemic species of *Phelsuma* in mainland Mauritius and *P. grandis* to determine whether they could share the same spatial distribution. We used Species Distribution Modelling (SDM) to predict the distribution of the endemic geckos and *P. grandis*, adjusted to their physical environment. SDM is widely used in studies of biogeography, global change ecology, conservation biology [19]–[22] and invasion biology [23]–[25], and is a valuable management tool [26]. We hypothesised that *P. grandis* is a “generalist” and so is able to occupy a broad climatic and habitat range which would overlap with the four endemic geckos. Second, we surveyed sites with and without *P. grandis* to determine their impact upon the number of endemic geckos. Abundance estimation in many ecological studies is based on counting the number of individuals seen without accounting for probability of detection of the species being studied [27]. Lack of detection can be affected by (i) site-level covariates such as behavioural changes in the presence of a competitor/predator and (ii) observation-level covariates such as climatic conditions, which might influence detection [27]–[29]. Hence, we used hierarchical binomial mixture modelling (BMM) [30] that implements a metapopulation approach to adjust abundance estimation to the probability of detection [31]. This enabled us to calculate the true abundance (hereafter, abundance) of geckos at each site. BMM has mainly been used in bird studies [32]–[33], but has also been used to estimate the abundance of amphibians [34]–[35] and mammals [36]. We hypothesised that *P. grandis* would have a negative impact on the abundance of the four endemic geckos. We then use this information to discuss the management of *P. grandis* in Mauritius and elsewhere.

## Methods

### Ethical statement

This study was approved by the University of Bristol's Ethical Review Committee (University Investigation Number UB/11/031) and the National Parks and Conservation Service, Ministry of the Agro-Industry, Mauritius.

### Predicting the distribution and overlap of endemic geckos and *P. grandis*

#### Collecting presence data

Articles were published in national newspapers requesting that members of the public report *P. grandis* sightings. We received more than 100 telephone calls giving locations of *P. grandis* across the island. Site visits were conducted to confirm their presence and geographical locations were recorded. Presence data on endemic species of *Phelsuma* were obtained through extensive field surveys in private and public forests between January and March 2010. Surveys were conducted along existing tracks between 07:00 and 19:00; any *Phelsuma* species seen were recorded. Opportunistic observations in urban areas were also collected between January 2010 and August 2012.

#### Environmental layers

We obtained 19 bioclimatic variables from WorldClim (http://www.worldclim.org/). They represented the annual trends in seasonality in Mauritius over the period 1950 to 2000 [37]. Elevation data were acquired from a Digital Elevation Model obtained from EOSDIS (http://reverb.echo.nasa.gov/r). We derived topographic information such as aspect and slope from the Digital Elevation Model. We also obtained Normalised Difference Vegetation Index (NDVI) data from a 2008 Landsat ETM+ dataset (http://earthexplorer.usgs.gov/).

All the variables were tested for multicollinearity using the pairwise Pearson's correlation test and only the most biologically meaningful ones with correlation values <0.7 were kept for further analyses. The nine retained variables were: BIO3 (isothermality), BIO4 (temperature seasonality), BIO7 (temperature annual range), BIO13 (precipitation of wettest period), BIO19 (precipitation of coldest quarter), aspect, elevation, NDVI and slope. As the bioclimatic variables had a resolution of 1 km^2^ and the other four layers had a spatial resolution of 0.0009 km^2^, we rescaled the five bioclimatic variables to 0.0009 km^2^ to take the advantage of the high resolution of the other layers to produce a map with 2,041,627 grid cells.

#### Model fitting and evaluation

Prior to analysis, we removed duplicate sightings of each species at the same or different sites within a grid cell. From the original 950 records, 777 presence data remained (*P. cepediana* 265; *P. grandis* 74; *P. guimbeaui* 174; *P. ornata* 220; *P. rosagularis* 44). We selected five algorithms to predict distribution and construct our ensemble models [38]: (i) Maximum Entropy Modelling (MaxEnt) [39]; (ii) Generalised Boosted Model (GBM) [40]; (iii) Random Forest (RF) [41]; (iv) Generalised Linear Models (GLMs) [42]; and (v) Generalised Additive Model (GAM) [43]; for model specifications for these algorithms see [44]. We implemented this particular approach because it is more accurate than using a single algorithm [38].

We randomly divided the original dataset, using 80% to construct the models and 20% to validate their accuracy. We carried out 10 repetition runs to obtain robust estimates of the species distributions, and model accuracy [45] was tested by means of the Area Under the Curve (AUC) of a Receiver Operating Characteristic plot (ROC) [46]. The AUC is a threshold independent measure of accuracy to evaluate the performance of SDMs [38], [47], [48]. Absence data were needed to evaluate model performance for all the algorithms except MaxEnt. Since only presence data were collected, we randomly generated background pseudo-absences i.e. locations with no sightings of a particular species were selected at random and assigned as absent. The number of pseudo-absences per species was ten times the number of sites with presence data [49].

**Figure 2:**
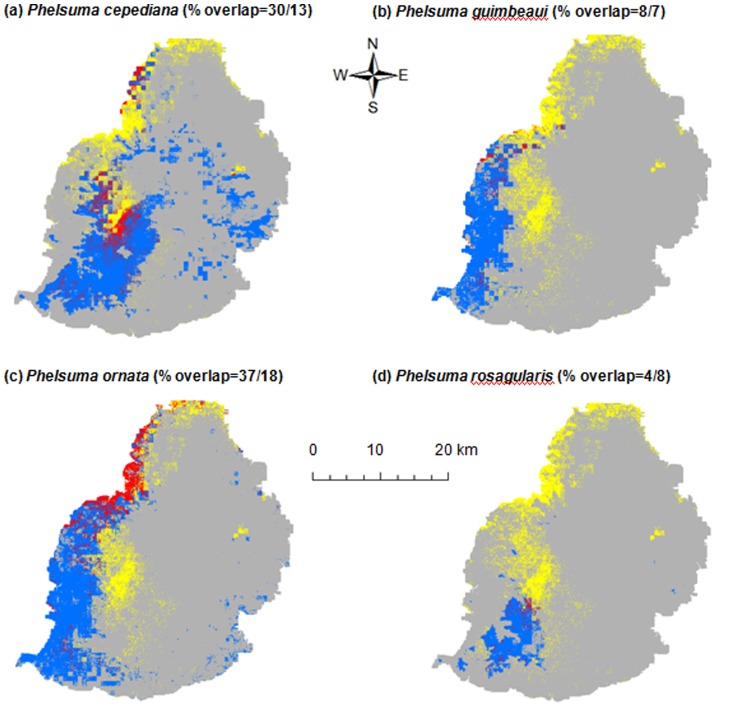
The binary projection and overlap between *Phelsuma grandis* and four endemic species of *Phelsuma*. Each map shows the overlap of *P. grandis* and one of the endemic species. On each map, grey indicates that no species of *Phelsuma* were predicted to be present, yellow shows the predicted range of *Phelsuma grandis*, blue the predicted range of that species of endemic *Phelsuma*, and red areas of predicted overlap between the two species. The first number in each heading is the % overlap of the predicted range of *Phelsuma grandis* with that species of endemic gecko and the second number is the % overlap of the endemic species' predicted range with that of *Phelsuma grandis*. The different maps suggest that *Phelsuma cepediana* (a) and *Phelsuma ornata* (c) will overlap more with *Phelsuma grandis* and thus could be at greater risk.

**Figure 3:**
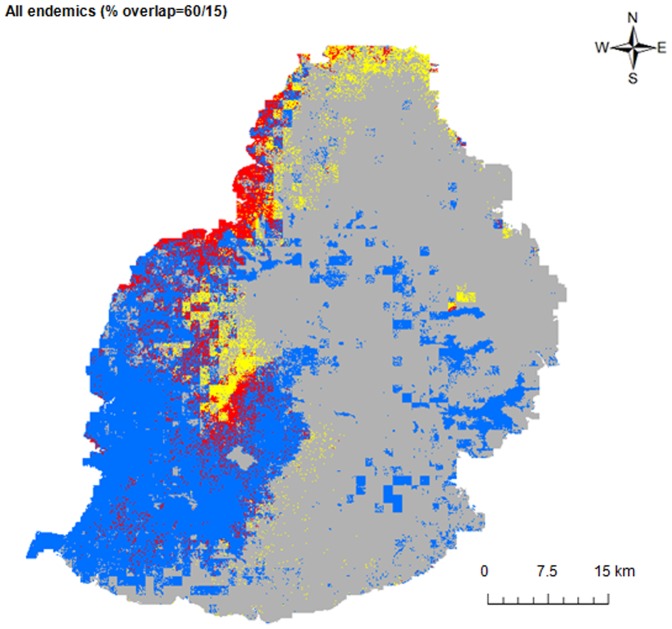
The binary projection and overlap between *Phelsuma grandis* and the combined predicted ranges of the four endemic species of *Phelsuma* on mainland Mauritius. Grey indicates that no species of *Phelsuma* were predicted to be present, yellow shows the predicted range of *Phelsuma grandis*, blue the combined predicted range of all the species of endemic *Phelsuma*, and red areas of predicted overlap. The first number in the heading is the % overlap of the predicted range of *Phelsuma grandis* with the combined predicted ranges of the four species of endemic *Phelsuma*, and the second number is the overlap of the endemic species' combined predicted ranges with the predicted range of *Phelsuma grandis*.

We considered an AUC<0.7 as a poor model and an AUC>0.9 as a highly accurate model [46]. We generated 50 models per species (i.e. 250 models in total) and models with an AUC>0.7 were selected to construct an ensemble model [44]. The ensemble model corresponded to the median probability of occurrence across the selected models for each grid cell. The median value was chosen because it was less sensitive to outliers than the mean. We converted the continuous predictions (Figure S1) into presence-absence prediction maps (Figures 2 and 3) to carry out further analyses. For this conversion, we applied an optimal probability threshold that maximises the sensitivity and specificity of the created models [50], [51]. The presence/absence map was used to project the potential distribution and overlap between each endemic species and *P. grandis*. We also calculated the predicted distribution and overlap between the combined range of the four species of endemic geckos and *P. grandis*. Since we considered *P. guimbeaui* and *P. rosagularis* to be the species most at risk, with only 30 and two known subpopulations respectively, we calculated the distance between the predicted range of *P. grandis* and known subpopulation of *P. guimbeaui* and *P. rosagularis*.

#### Niche breadth

We examined the environmental breadth indices to determine whether *P. grandis* had a narrow (specialist = 0) or wide (generalist = 1) niche. We used the binary prediction of presence and absence maps (Figures 2 and 3), modelled from the combined influence of all the environmental predictors and occurrence of each species, to estimate niche breadths. We used Levins' niche breadth metrics [52], where a value of 0 was equivalent to only one grid cell being suitable and a value of 1 was equivalent to all grid cells being suitable [53].

#### Data handling

Data processing, model fitting and projection were conducted in R (R 3.0.1 Development Core Team 2013) BIOMOD2 package [44], while breadth metrics were estimated in ENMTools (http://enmtools.blogspot.co.uk/). SDM predicted range and overlap estimation and GIS manipulations were carried out in ARCGIS 10.1 (ESRI, Redlands, California, USA) and Quantum GIS 1.8 (http://www.qgis.org/en/site/).

### Impact of *P. grandis* on the abundance of endemic geckos

#### Field surveys

We conducted visual estimate surveys (VES) at ten sites where *P. grandis* were present and 11 sites where they were believed absent (Figure 1). We used two types of categorical habitat data: (i) building or non-building and (ii) vegetation type i.e. palm or non-palm. There were four building sites (two with, two without *P. grandis*), four non-palm sites (two with, two without *P. grandis*) and 13 palm sites (six with, seven without *P. grandis*). With the exception of one palm site, each *P. grandis* site was matched with a site without *P. grandis* that had comparable habitat characteristics in terms of area, number of trees and tree diameter at breast height (DBH). Building sites were human dwellings in residential areas along the south-east coast of Mauritius, with an average area of 176 m^2^ and 189 m^2^ for sites with and without *P. grandis*. Palm sites contained trees from the Arecaceae family. Each consisted of an isolated clump of four to 12 palm trees with a height less than 12 m and occupying an area from 50 m^2^ to 100 m^2^. Average basal tree coverage (based on DBH) was 0.26 m^2^ and 0.21 m^2^ in sites with and without *P. grandis*. Non-palm sites were isolated and covered an area of ∼400 m^2^ with a maximum of four trees up to 20 m high, mainly *Terminalia arguna* (terminalia) and *Ficus benghalensis* (banyan tree). The average basal tree coverage was 10.12 m^2^ and 10.00 m^2^ in sites with and without *P. grandis*.

To calculate the abundance of endemic geckos, one person slowly walked round each site, scanning every tree and wall surface, counting all the geckos seen and identifying them to species. We used features such as bite marks, size and general colour patterns to avoid repeat counts of the same gecko. This was repeated for 15 minutes each hour during peak activity periods from 08:00 to 11:00 and 14:00 to 18:00, i.e., six times in a day. All 21 sites were surveyed for one day between 16 and 23 May 2011. We assumed that the different time slots and minimum distance between sites (>100 m) would ensure that the sites would be temporally and spatially independent. Temperature was recorded at the start of each count using a Lutron Lm-8000 environmental meter (Lutron Electronic Enterprise Co. Ltd., Taipei, Taiwan). Cloud cover was estimated visually to the nearest 5%. Temperature and cloud cover were included as observation-level covariates to test their respective effects on detection probabilities. Site-level covariates, such as status (presence or absence) of *P. grandis* and habitat types were modelled to investigate their effects on abundance.

#### Data analyses

The emergence of hierarchical models has decreased the dependence on labour-intensive mark recapture in the estimation of population parameters such as abundance and occupancy [54]. Simulation studies have demonstrated that BMM was robust in the estimation of abundance [55], but dependent upon meeting key assumptions i.e. that the population is closed during the survey and that the sites are spatially and temporally independent [56].

We developed a BMM using two probability distributions:-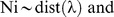


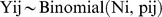
where Ni = the abundance of geckos in site i; dist = either Poisson, negative binomial or zero-inflated Poisson distribution; λ = mean gecko abundance; Yij = number of geckos recorded at site i during survey j; pij = probability of detecting any gecko at site i during survey j; i = site 1,2,…21; and j = the number of the survey 1,2…6.

An integrated likelihood method as implemented in the unmarked R package [57] was used to calculate the above variables. We found the most parsimonious model by an information-theoretic approach using the corrected Akaike Information Criterion (AICc) in the R package AICcmodavg [58]. The AICc was used because the sample size (n) divided by the number of parameters (K) was less than 40 [59]. Model adequacy of the global model was tested with a parametric bootstrapping chi-squared goodness of fit. Depending on the model adequacy, a Poisson, negative binomial or zero-inflated Poisson probability distribution was used to determine abundance. Only the top model was used to predict the average detection probability and abundance under different covariate conditions.

## Results

### Species distribution modelling and niche breadths

Only 9 of the 250 SDMs i.e. 7 GLMs, 1 MaxEnt and 1 GAM had an AUC<0.7 and were therefore excluded from the ensemble model. Four ensemble models were considered as excellent with AUC>0.9 and one as a good model with AUC>0.8 (Table 1). *P. cepediana* had the largest predicted suitable range of 376 km^2^ (Table 1, Figure 2a). *P. grandis* had a predicted suitable range of 161 km^2^ in the centre and along the west to the north-east coast of Mauritius (Table 1, Figure 3). *P. guimbeaui* had a predicted suitable range of 186 km^2^ on the west side of the island (Table 1, Figure 2b). *P. ornata* had a predicted suitable range of 342 km^2^ along the coast, particularly from the south-west to north-west (Table 1, Figure 2c). *P. rosagularis* had the smallest predicted suitable range, only 85 km^2^, mainly in south-west Mauritius (Table 1, Figure 2d).

**Table 1:** Ensemble model evaluation results, showing the Area Under the Curve (AUC) of the median ensemble model for the five species of *Phelsuma*.

Species	AUC	Threshold	Sensitivity	Specificity	Predicted range (km^2^)
*P. cepediana*	0.888	0.438	84.9	78.3	376
*P. grandis*	0.973	0.566	98.3	90.1	161
*P. guimbeaui*	0.960	0.568	89.2	91.1	186
*P. ornata*	0.951	0.309	97.3	80.7	342
*P. rosagularis*	0.995	0.515	100.0	95.7	85

*Phelsuma cepediana* had a good model fit (AUC>0.8), while the other four species had an excellent model fit (AUC>0.9). A probability threshold was used to maximise sensitivity and specificity to produce presence/absence maps to predict the range of each species.

Between 4% and 37% of the predicted suitable range of *P. grandis* overlapped with each endemic species, whereas 7% to 18% of the predicted suitable range of each endemic species overlapped with *P. grandis* (Figure 2). For *P. grandis*, 60% of its predicted range overlapped with the combined area of suitability for all four endemic species of *Phelsuma*, whereas 15% of the predicted ranges of all the four endemic species overlapped with *P. grandis* (Figure 3). Of the 32 known subpopulations of *P. guimbeaui* and *P. rosagularis*, 13 were within the predicted range of *P. grandis*, four within 50 m, five within 200 m, five within 500 m, three within 1000 m and two within 1500 m.

Niche breadth for the five species of *Phelsuma* varied from 0.140 to 0.652. *P. guimbeaui* and *P. rosagularis* had a narrow specialist niche breadth metric, whereas *P. cepediana*, *P. grandis* and *P. ornata* had a moderate to broad generalist niche breadth metric (Table 2).

**Table 2:** Levins' niche breadth metric, where restricted to specific environmental conditions (specialist) = 0 and able to exploit a wide range of environmental conditions (generalist) = 1.

Species	Levins' niche breadth
*P. cepediana*	0.652
*P. grandis*	0.497
*P. guimbeaui*	0.179
*P. ornata*	0.356
*P. rosagularis*	0.140

### Effect of *P. grandis* on the abundance of endemic geckos

We recorded three endemic species, *P. cepediana*, *P. guimbeaui* and *P. ornata*, during visual surveys of the 21 sites. Since endemic geckos were sympatric, and of similar sizes and highly detectable colours, we combined their counts and compared abundance adjusted to imperfect detection probability between sites with and without *P. grandis*. On 126 surveys (21 sites each surveyed 6 times), we recorded 32 sightings of endemic geckos and 346 of *P. grandis* in the invaded sites, and 441 sightings of endemic geckos and no *P. grandis* in the control sites. We only detected endemic geckos in six of the ten sites with *P. grandis*.

We used three different statistical distributions to test for over-dispersion. The Poisson distribution showed signs of over-dispersion in the global model and was not used in model fitting. The negative binomial and zero-inflated Poisson distributions passed the goodness of fit test and adequately fitted the abundance model; the zero-inflated Poisson distribution for abundance was selected because it had smaller confidence intervals.

**Figure 4:**
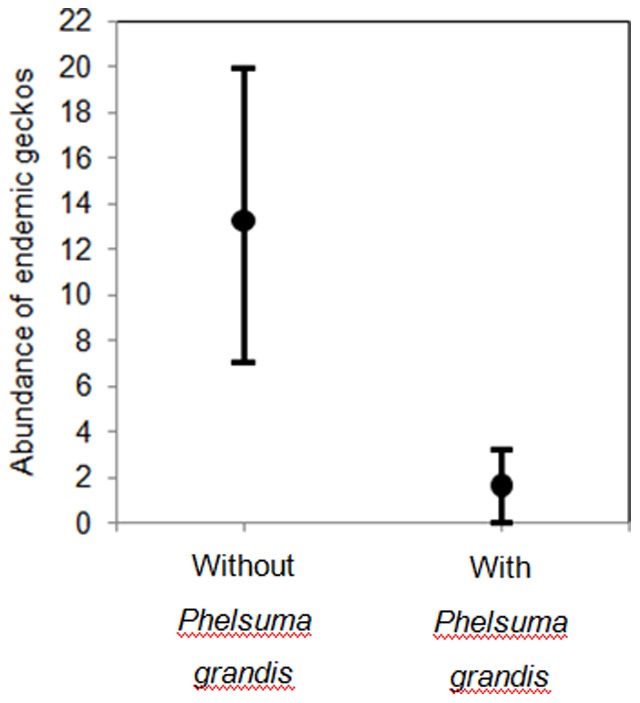
The mean endemic gecko abundance (with 95% confidence intervals) in sites with and without *Phelsuma grandis*. Sites without *Phelsuma grandis* had a high abundance of endemic geckos, those with *Phelsuma grandis* a low abundance of endemic geckos. N = 10 sites with *Phelsuma grandis*, N = 11 sites without *Phelsuma grandis*.

The lowest AICc model (AICc weight of 76%) was chosen as the best fitting model (Table 3). The selected model showed that the abundance of endemic geckos was affected by the presence of *P. grandis*; the abundance of endemic geckos was 13.3 (95% confidence interval 7.0–19.9) and 1.6 (95% confidence interval 0.0–3.2) in sites without and with *P. grandis* respectively (Figure 4). This represented a decline of 89% of endemic geckos in *P. grandis* sites.

**Table 3:** Model selection results. Abundance was modelled with habitat and status as site-level covariates.

Detection	Abundance	K	AICc	Delta AICc	AICc weight
Cloud+habitat	Status	7	477.4	0.0	0.76
Cloud+status	Status	6	482.0	4.6	0.07
Cloud+habitat+status	Status	8	482.6	5.3	0.05
Habitat+status	Status	7	483.3	5.9	0.04
No covariate	Status	4	484.1	6.8	0.03
Status	Status	5	487.6	10.2	0.00
Habitat+status+temp	Status	8	488.4	11.0	0.00
Cloud+habitat+status+temp	Status	9	488.6	11.2	0.00
Cloud+habitat+status+temp	Habitat+status	11	490.3	12.9	0.00
Cloud	Habitat	6	527.2	49.9	0.00
Habitat	Habitat	7	527.9	50.5	0.00
Cloud+temp	Habitat	7	530.3	52.9	0.00
Cloud+habitat+temp	Habitat	9	531.8	54.4	0.00
Habitat+status	Habitat	8	533.3	55.9	0.00
Cloud+habitat	No covariate	6	537.4	60.0	0.00
Status	Habitat	6	539.0	61.7	0.00
Habitat	No covariate	5	540.9	63.6	0.00
No covariate	No covariate	3	549.9	72.5	0.00
Status	No covariate	4	552.8	75.4	0.00

K = number of parameters used.

Delta AICc = difference between lowest AICc model and model AICc.

AICc weight = model probability among all candidate models.

Detection probability was modelled with observation-level covariates: cloud = cloud percentage cover; habitat = building, non-palm or palm; status = presence or absence of *P. grandis*; and temp = temperature. We used the corrected Akaike Information Criterion (AICc) to determine the best supported model.

**Figure 5:**
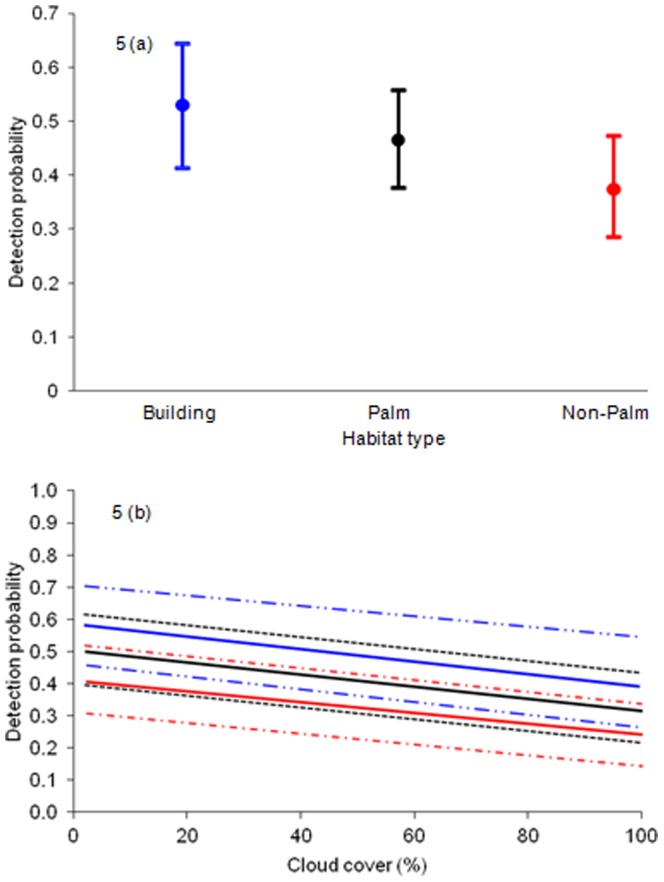
Effects of habitat type and cloud cover in each habitat type on detection probability. Figure 5a shows that the detection probability (with 95% confidence intervals) was similar in the three habitat types, with detection probability slightly higher in building sites than palm followed by non palm sites. Figure 5b shows a general decrease in detection probability (with 95% confidence intervals indicated by broken lines) with an increase in cloud cover in the three habitat types. Blue indicates building sites, black palm sites and red non-palm sites.

Probability of detection was affected by habitat and cloud cover (Table 3). Probability of detection was similar in the three habitats, with the highest detection in building sites (0.530, 95% confidence interval 0.413–0.644), followed by palm (0.465, 95% confidence interval 0.375–0.557) and non-palm sites (0.374, 95% confidence interval 0.284–0.437) (Figure 5a). This is not surprising: building sites had the least vertical diversity, making it relatively easy to spot geckos. The higher detectability between palm versus non-palm sites can be explained by the simple structure of palms with heights less than 12 m, compared to the complex structure of branching non-palm trees with heights up to 20 m. An increase in cloud cover had a negative effect on probability of detection in the three habitat types because the geckos do not bask in cloudy conditions, and so are less likely to be detected as they find refuge in optimal thermo-regulatory spots such as between leaves (Figure 5b).

## Discussion

We used SDMs combined with BMM to investigate the ecological impacts of *P. grandis* on the endemic *Phelsuma* community in mainland Mauritius. The large AUC values for the five ensemble models suggest that the predictive maps had high statistical robustness. Though SDMs can give an accurate prediction of the spatial distributions of IAS [24], interpretation of the results need to be treated with caution. Model projections assumed complete random dispersal. However, as gecko movements are restricted by habitat isolation and fragmentation, the predictive maps of all five species could be overestimates [60]. Certainly, for both *P. guimbeaui* and *P. rosagularis*, there are very few known subpopulations within their predicted range. Some studies advocate the use of variables such as species interactions [61], species traits [62] or “natural history” [63] to build SDMs. However, little information was available for these variables, especially for *P. grandis*. BMM model performance using the zero-inflated Poisson distribution showed that our models were robust and had a good fit to the data. We used BMM to account for imperfect detection in the estimation of the abundance of geckos [34], [36], [57], [64], since this can lead to erroneous conclusions [27], especially when invasive and native species co-occur [65]. *Phelsuma* spp. tend to partition their habitats along an axis to reduce competition [66], [67], and so one possible response would be for the endemic species to shift along an axis following invasion by *P. grandis*. However, we did not observe any shift in habitat selection by endemic geckos in the presence of *P. grandis*; there were significantly fewer endemic geckos in the presence of *P. grandis*, and endemic geckos were not even detected at four of the ten sites with *P. grandis*. Despite being a snapshot survey, we believe that the same results would have been observed throughout the year.

### Characteristics of a generalist invader and the potential threat of invasion

The predictive overlap maps and a relatively large niche breadth suggest that *P. grandis* has the typical attributes of a generalist invader [68], with the ability to persist in a large range of environmental conditions. The predictive maps also suggest that the two commonest species, *P. cepediana* and *P. ornata*, are the most threatened by *P. grandis*. However, the distance between the predicted range of *P. grandis* and known subpopulations of *P. guimbeaui* and *P. rosagularis* were relatively small, suggesting that they could be under more immediate threat from *P. grandis* invasion. Both these endemic species had narrow Levins' niche breadths typical of specialists; they also occupy small restricted ranges such that the potential threat posed by *P. grandis* enhances their vulnerability to extinction [7].

With a relatively large niche breadth, we expected *P. grandis* to have a bigger predicted distribution. For example, the niche breadth of *P. ornata* was 0.356, with a predicted range of 342 km^2^, compared to a niche breadth of 0.497 and a predicted range of 161 km^2^ for *P. grandis*. One plausible explanation for this apparent disparity is that our presence data do not reflect the full extent of the niche suitability for *P. grandis*, which has not had time to invade all the available niches in the 20 years since its introduction. For instance, in its native range in Madagascar (approximately 1000 km to the west of Mauritius), *P. grandis* has been recorded at elevations up to 900 m [15]. This suggests that the whole altitudinal range of Mauritius, which has a maximum altitude of 828 m, is vulnerable to invasion. However, we had no records of *P. grandis* above 700 m in Mauritius. When an IAS first arrives, it usually utilises habitats similar to its native range [69] and subsequently expands and invades new niches [9], [70]. We suspect that eventually *P. grandis* will have a larger range in Mauritius than we have predicted from its early spread and pattern of habitat selection, and so there will be more extensive overlap with the ranges of the endemic species of *Phelsuma*.

### The threat to endemic geckos

There was a dramatic decline or total absence of endemic species of *Phelsuma* in the presence of *P. grandis*, suggesting that extirpation follows the arrival of *P. grandis*. Observations by local residents near the study sites suggest that *P. grandis* took less than 12 years to cause the disappearance of the endemic species. Similarly, reports from the public in Baie du Tombeau indicate that, in the two decades since its release, *P. grandis* has colonised the entire 1.8 km^2^ suburban region and no *P. cepediana*, *P. guimbeaui* or *P. ornata* have been seen in this area since 2009.

How *P. grandis* is leading to the extirpation of endemic *Phelsuma* populations is unclear, although competitive exclusion and predation are believed to be key drivers of species extinction [71], [72]. Field observations suggest that *P. grandis* shares temporal and spatial niches with the endemic geckos and so there is potential for competition and predation. Being larger than the endemic geckos, *P. grandis* is likely to consume larger prey items and thus relax competition for food [73], although day geckos frequently feed on nectar, pollen [74] and tree saps, and so there may be competition with *P. grandis* for these food resources [75]. We often observed aggressive behaviour from *P. grandis* towards endemic geckos at inflorescences and tree-sap foraging sites, suggesting competitive displacement at important food resources. A decrease in egg production [76], [77] and/or reduced mating success [77] can occur in the presence of a predator or competitor, leading to a decline in reproductive output and increasing the risk of extinction. Understanding the underlying causes of how *P. grandis* leads to the decline and local loss of endemic *Phelsuma* populations will be key to quantifying the long-term impact and potentially managing systems to mitigate the threats.

*P. grandis* has also been introduced in nearby Réunion. Two endemic species of day gecko, *P. borbonica* and *P. inexpectata* (Manapany day gecko), are both threatened by the arrival of *P. grandis* [18]. *P. grandis* is widely available in the pet trade and there is the risk of further invasions in countries such as the Comores and Seychelles that have their own endemic species of *Phelsuma*.

## Conclusions

With the ever-increasing numbers of invasive species, it is important to decide whether an IAS needs to be eradicated. Ideally, this decision needs to be made before the IAS is well-established. While the eradication of an IAS can help in the restoration of native ecosystems [78], eradication can also cause more damage [79], especially when an IAS has established key ecological functions [9]. However, since the majority of alien species will never be eradicated, alternative management strategies may be more appropriate in many circumstances [80].

*P. grandis* is a relatively recent introduction to Mauritius. Our data suggest that it is a generalist capable of invading a diversity of Mauritian habitats with dramatic impacts on the endemic *Phelsuma* community. Currently, *P. grandis* is localised in Mauritius, and mostly occurs in private gardens and plantations. However, this poses additional risks since it could be spread by accidental anthropogenic transportation. *P. grandis* is also being actively moved to new locations by locals to control other introduced geckos that are considered to be a messy and noisy nuisance in houses. It is now critical to identify the mechanisms whereby *P. grandis* leads to the loss of local populations of endemic *Phelsuma* to limit further impacts, and to decide if, and how, an eradication programme could be undertaken before *P. grandis* becomes better established in Mauritius. Our data also highlight the importance of banning the importation of *P. grandis* to other countries, particularly those with vulnerable populations of *Phelsuma* and other endemic geckos.

## Supporting Information

Figure S1 **The continuous probability of occurrence of the five species of** ***Phelsuma*** **using the ensemble model with the highest probability of suitability indicated by red and the lowest by grey.**
(TIF)
