# Supplementary figures and images for: Ecological Effects of the Invasive Giant Madagascar Day Gecko on Endemic Mauritian Geckos: Applications of Binomial-Mixture and Species Distribution Models
Source: PLoS One. 2014 Apr 30;9(4):e88798. doi: 10.1371/journal.pone.0088798 (PMC4005729; doi:10.1371/journal.pone.0088798)

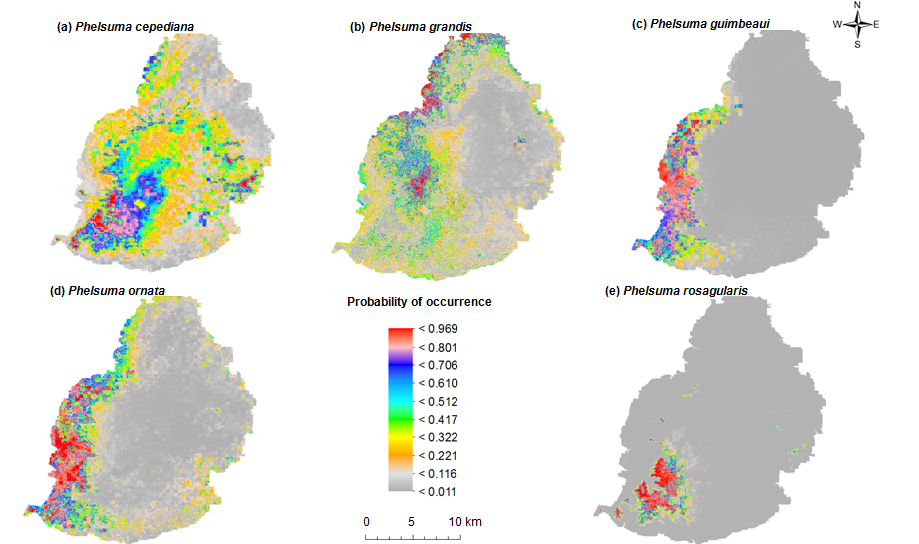

Supplement: Figure S1 — The continuous probability of occurrence of the five species of Phelsuma using the ensemble model with the highest probability of suitability indicated by red and the lowest by grey. (TIF) [file pone.0088798.s001.tif]
